# Within-Subject Correlation Analysis to Detect Functional Areas Associated With Response Inhibition

**DOI:** 10.3389/fnhum.2018.00208

**Published:** 2018-05-22

**Authors:** Tomoko Yamasaki, Akitoshi Ogawa, Takahiro Osada, Koji Jimura, Seiki Konishi

**Affiliations:** ^1^Department of Neurophysiology, Juntendo University School of Medicine, Tokyo, Japan; ^2^Department of Biosciences and Informatics, Keio University School of Science and Technology, Yokohama, Japan; ^3^Research Institute for Diseases of Old Age, Juntendo University School of Medicine, Tokyo, Japan; ^4^Sportology Center, Juntendo University School of Medicine, Tokyo, Japan

**Keywords:** human, functional magnetic resonance imaging, cognitive control, executive function, performance

## Abstract

Functional areas in fMRI studies are often detected by brain-behavior correlation, calculating across-subject correlation between the behavioral index and the brain activity related to a function of interest. Within-subject correlation analysis is also employed in a single subject level, which utilizes cognitive fluctuations in a shorter time period by correlating the behavioral index with the brain activity across trials. In the present study, the within-subject analysis was applied to the stop-signal task, a standard task to probe response inhibition, where efficiency of response inhibition can be evaluated by the stop-signal reaction time (SSRT). Since the SSRT is estimated, by definition, not in a trial basis but from pooled trials, the correlation across runs was calculated between the SSRT and the brain activity related to response inhibition. The within-subject correlation revealed negative correlations in the anterior cingulate cortex and the cerebellum. Moreover, the dissociation pattern was observed in the within-subject analysis when earlier vs. later parts of the runs were analyzed: negative correlation was dominant in earlier runs, whereas positive correlation was dominant in later runs. Regions of interest analyses revealed that the negative correlation in the anterior cingulate cortex, but not in the cerebellum, was dominant in earlier runs, suggesting multiple mechanisms associated with inhibitory processes that fluctuate on a run-by-run basis. These results indicate that the within-subject analysis compliments the across-subject analysis by highlighting different aspects of cognitive/affective processes related to response inhibition.

## Introduction

In human fMRI studies, brain activity is generally used to identify functional areas associated with brain functions. Brain-behavior correlation is often used to detect functional areas, calculating correlation between the behavioral index and the brain activity related to a particular function in the group level. In the case of the stop-signal task (Logan and Cowan, 1984; Rubia et al., 2001; Aron et al., 2003), a standard task to probe response inhibition, the correlation is calculated between the stop-signal reaction time (SSRT) and the brain activity related to response inhibition. Previous studies have revealed functional areas related to the response inhibition, including the inferior frontal cortex, the pre-supplementary motor area, the superior frontal cortex, the anterior cingulate cortex, the striatum, the subthalamic nucleus and the cerebellum (Aron and Poldrack, 2006; Garavan et al., 2006; Li et al., 2006; Aron et al., 2007; Forstmann et al., 2008, 2012; Congdon et al., 2010; Rubia et al., 2010; Boehler et al., 2011; Ghahremani et al., 2012; Hirose et al., 2012; Jimura et al., 2014).

**Figure 1:**
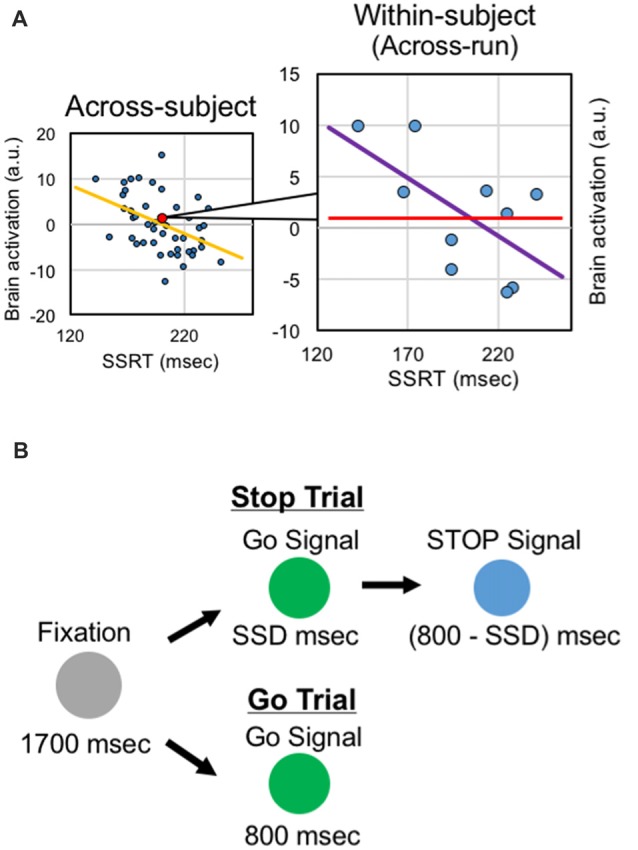
Analysis and task design. **(A)** Within-subject correlation analysis. In the across-subject analysis, correlation between the behavioral index and the brain activity is calculated across subjects. In the within-subject analysis, correlation between the behavioral index and the brain activity is calculated across runs within the same subjects. **(B)** The stop-signal task. The task consisted of the go trials and stop trials, presented in different colors. In the stop trials, after presentation of the stop signal, subjects were instructed to stop manual responses.

These previous studies of response inhibition calculated the brain-behavior correlations across subjects, regarding data from one subject as one sample for the correlation analysis, based on inter-individual variability. It is also possible to utilize intra-individual variability of executive functions, instead of inter-individual variability, and to calculate correlation across fMRI runs of the same subjects, regarding data from one run of the same subject as one sample for the correlation analysis (Figure 1A). Such analyses have been conducted in a trial basis (e.g., Christoff et al., 2001; Yarkoni et al., 2009). It is to be noted, however, that the SSRT is estimated, by definition, not in a trial basis but from pooled trials such as fMRI runs (Logan and Cowan, 1984; Verbruggen et al., 2013). The within-subject analysis may complement the results from the across-correlation analysis by focusing on cognitive fluctuations in a shorter time period. However, despite the abundant literatures reporting brain-behavior correlation based on the across-subject analysis, very little about response inhibition has been reported based on the within-subject analysis. More broadly, neural mechanisms of learning response inhibition have been studied, mostly tracking time courses of brain activity (Toni et al., 2001; Milham et al., 2003; Kelley et al., 2006; Erika-Florence et al., 2014; Berkman et al., 2014; Hampshire et al., 2016). However, time-related changes of the within-subject correlations have rarely been examined.

In this study, we conducted the within-subject correlation analysis using the data published in a study of the across-subject correlation applied to the stop-signal task (Jimura et al., 2014). Correlation between the SSRT and the brain activity related to response inhibition was calculated based on the across- and within-subject analyses (Figure 1A), and the results from both of them were compared. We also examined the time-dependent changes of the within-subject correlation using the same dataset, based on comparison between earlier and later runs as conducted previously (Jimura et al., 2014).

## Materials and Methods

### Subjects

The present study reanalyzed the data published previously (Jimura et al., 2014). Forty-six healthy right-handed subjects (26 males, 20 females; age range: 20–26) participated in this study. This study was carried out in accordance with the recommendations of the guideline regarding the ethics of noninvasive research of human brain functions by Japan Neuroscience Society with written informed consent from all subjects. All subjects gave written informed consent in accordance with the Declaration of Helsinki. The protocol was approved by the institutional review board of Juntendo University School of Medicine.

### Imaging Procedures

The imaging procedures are described previously in more detail (Jimura et al., 2014). The experiments were conducted using a 3.0 T-MRI system. T1-weighted structural images were then obtained for anatomical reference (76 × 2-mm slices; in-plane resolution: 1 × 1 mm). For functional imaging, a gradient echo echo-planar sequence was used (40 × 4-mm slices; TR = 3000 ms; TE = 50 ms; flip angle = 90 degree; in-plane resolution: 4 × 4 mm). Each functional run consisted of 64 whole-brain acquisitions. Twelve functional runs were administered for each subject.

### Behavioral Procedures

The behavioral procedures are described previously in more detail (Jimura et al., 2014). Subjects performed a stop-signal task (Logan and Cowan, 1984). The stop-signal task is depicted in Figure 1B. At the beginning of the trial, a gray circle was presented for 1700 ms. In the GO trial, then, a green circle was presented for 800 ms, and the subjects were instructed to make a button press with the right thumb. In the STOP trial, a green circle was presented. After a stop-signal delay (SSD), the green circle was changed to a blue circle, and the subjects were required to withhold the manual response. The color of Go signal and Stop signal was counterbalanced across subjects. The SSD was updated on each STOP trial based on a tracking procedure, allowing us to maintain accuracy of the STOP trial at approximately 50% (Band et al., 2003).

To evaluate the efficiency of the response inhibition, this study estimated a behavioral index, SSRT for each subject based on an integration method (Logan and Cowan, 1984; Verbruggen et al., 2013). SSRT is a behavioral index reflecting the response inhibition efficiency, and individuals with shorter SSRTs can be considered as more efficient in response inhibition (Logan and Cowan, 1984). Each functional run contained 16 STOP trials and 48 GO trials (STOP/GO ratio = 1:3). Each subject underwent a total of 12 runs.

### Data Analysis

The brain activity related to response inhibition was examined in the same way as the previous study (Jimura et al., 2014). Functional images were preprocessed using SPM8^1^. The images were first realigned, then corrected for slice timing, and spatially normalized to a standard MNI template with interpolation to a 2 × 2 × 2 mm space, followed by spatial smoothing with an 8-mm kernel. Events of interest (GO success and STOP success), together with nuisance events (GO fail and STOP fail), were coded at the onset of the GO signal of each trial and were modeled as transient events in a general linear model. Single-level analysis was performed to estimate signal magnitudes, and the magnitude images were contrasted between STOP success and GO success trials in the 3rd to 12th runs, during which SSD, SSRT and accuracy of STOP trials were found stable (Jimura et al., 2014). Group-level statistics were estimated in a one-sample *t*-test, treating subjects as a random effect.

As a positive control, the across-subject brain-behavior analysis was performed to replicate the results reported previously (Jimura et al., 2014). The voxel-wise correlation was calculated between the SSRT and the signal magnitudes for the contrast STOP success minus GO success during the stable runs (i.e., 3rd to 12th runs). The correlation coefficient was then converted to Fisher’s z, and the Fisher’s z was further normalized to a z gaussian distribution to indicate statistical significance level.

Within-subject brain-behavior analysis was also performed, calculating the correlation between the SSRT and the signal magnitudes for the contrast STOP success minus GO success for each run in the stable runs (i.e., 3rd to 12th runs) of the same subjects. The correlation coefficient for each subject was then converted to Fisher’s z, and the Fisher’s z was entered into a one-sample group-mean test, treating subjects as a random effect. To correct for multiple comparisons, statistical testing was performed based on non-parametrical permutation inference (Eklund et al., 2016) implemented in *randomise* in FSL suite (Winkler et al., 2014,^2^). Cluster-wise statistical correction was performed for voxel clusters defined by a threshold (*P* < 0.01, uncorrected; Eklund et al., 2016), and then significance level was assessed above *P* < 0.05 corrected for multiple comparisons within a functional areas associated with response inhibition identified by meta-analysis of forward inference in Neurosynth^3^ (Yarkoni et al., 2011) for cortical areas, and also across the whole brain for other brain areas.

To examine the temporal changes in correlations, the data set (3rd to 12th runs) was divided into two parts. To keep the minimal number of samples for the within-subject correlation analysis, the first six runs (3rd to 8th runs) and the last six runs (7th to 12th runs) were classified into FIRST and SECOND, with the middle 7th and 8th runs doubled in the two parts. Unlike Jimura et al. (2014) where 46 subjects could be used for the across-subject correlation analysis of FIRST and SECOND, 10 runs had to be divided for the within-subject correlation analysis of FIRST and SECOND in the present study. We ameliorated this issue by duplicating two runs (7th–8th runs): 3rd–8th for FIRST and 7th–12th for SECOND. However, further duplication (6th–9th runs) will not be acceptable because, in the case of the 3rd–9th runs for FIRST and 6th-12th runs for SECOND, more than half of the data points (four out of seven data points) will be doubled. So, we chose minimal duplication to ameliorate statistical power. Then the across- and within-subject analyses were performed between the SSRTs and activation magnitudes, and the two correlation maps (FIRST and SECOND) were Fisher’s z-transformed and were normalized to a z gaussian distribution.

## Results

### Behavioral Results

**Figure 2:**
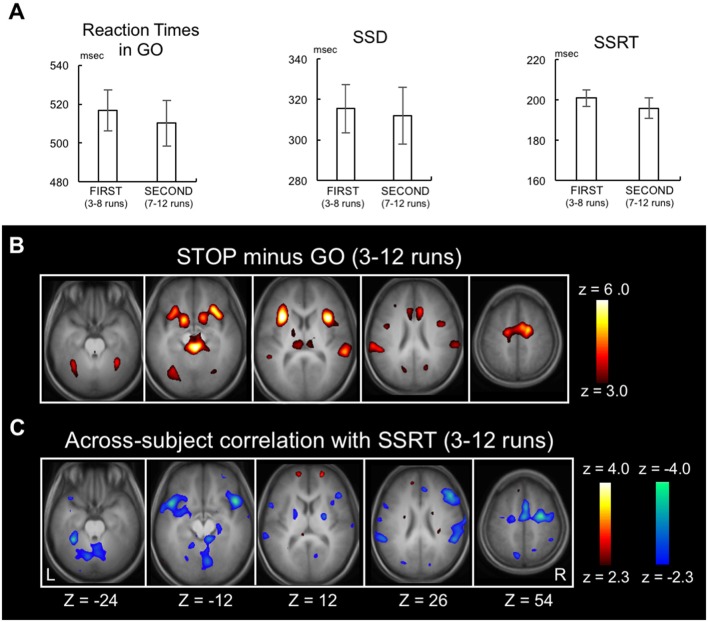
Replication of previously published data. **(A)** Go reaction time, stop signal delay and the stop-signal reaction time (SSRT) in FIRST (3rd to 8th) and SECOND (7th to 12th) parts of the runs. Error bars indicate standard error of means. **(B)** Statistical maps of brain activation during response inhibition in the whole stable runs (3rd to 12th) revealed by the contrast Stop success trials vs. Go success trials. The color scale reflects statistical significance as shown by the color bar to the right (above *z* > 2.3 for a display purpose). Z below the statistical maps indicates the Z coordinate in MNI atlas. **(C)** Statistical maps of the across-subject correlation between the SSRT and the brain activity related to response inhibition in the whole stable runs (3rd to 12th).

Behavioral results were shown for the 3rd to 12th runs, during which SSD, SSRT and accuracy of STOP trials were found stable (Jimura et al., 2014). The RT of GO trials, SSD and SSRT in the 10 runs were 514.3 ± 73.2 ms (mean ± SD), 314.2 ± 84.9 ms and 197.8 ± 30.9 ms, respectively. The differences between FIRST (3rd to 8th runs) and SECOND (7rd to 12th runs) were not significant in any of these behavioral measures (*P* = 0.12, *P* = 0.53, *P* = 0.07, respectively; Figure 2A), suggesting that behavioral efficiency of response inhibition was constant between these periods.

### Imaging Results

As a positive control, the brain activity during STOP success relative to GO success during the stable period (3rd to 12th runs) was calculated (Figure 2B). Although the same authors conducted the analysis, there existed slight differences from Jimura et al. (2014) regarding the brain activation and the across-subject correlation, presumably due to differences in update versions of OS (MS Windows), Matlab and SPM8. However, as reported previously, activations were observed in multiple areas including the inferior frontal gyrus, pre-supplementary motor area, and temporo-parietal junction and anterior insula (Konishi et al., 1998, 1999; Garavan et al., 1999; de Zubicaray et al., 2000; Liddle et al., 2001; Menon et al., 2001; Rubia et al., 2001; Bunge et al., 2002; Durston et al., 2002a,b; Mostofsky et al., 2003; Hester et al., 2004; Kelly et al., 2004; Matsubara et al., 2004; Brass et al., 2005; Aron and Poldrack, 2006; Chambers et al., 2006, 2009; Li et al., 2006, 2008; Leung and Cai, 2007; Sumner et al., 2007; Nakata et al., 2008; Zheng et al., 2008; Cai and Leung, 2009; Chao et al., 2009; Chikazoe et al., 2009a,b; Sharp et al., 2010; van Gaal et al., 2010; Zandbelt and Vink, 2010; Boecker et al., 2011; Arbula et al., 2017). Correlations were also calculated between the SSRTs and the brain activity (STOP minus GO) in the 3rd to 12th runs (Figure 2C, see Supplementary Figure S1 for whole-brain slices). Negative correlations were observed in cortical, subcortical and cerebellar regions, consistent with prior studies (Li et al., 2006, 2008; Aron et al., 2007; Congdon et al., 2010; Boehler et al., 2011; Ghahremani et al., 2012; Hirose et al., 2012).

**Figure 3:**
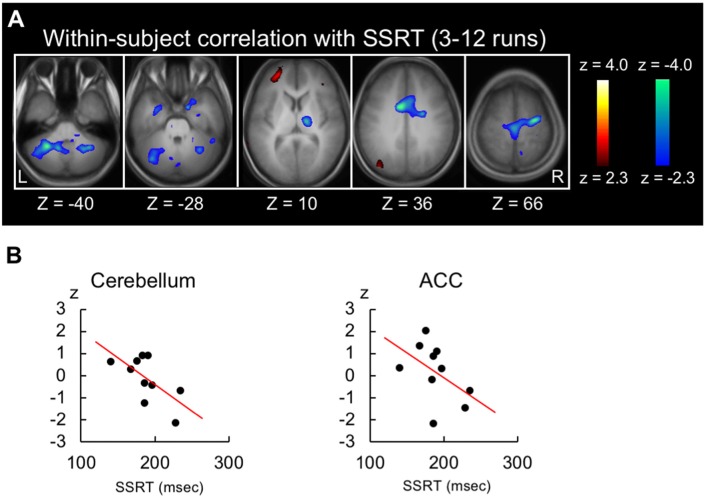
Results of the within-subject correlation analysis. **(A)** Statistical maps of the within-subject (across-run) correlation between the SSRT and the brain activity related to response inhibition in the whole stable runs (3rd to 12th). The format is similar to that in Figure 2C. **(B)** Scatter plots of the within-subject correlation in the anterior cingulate and cerebellar regions for one representative subject.

Because the shorter SSRT indicates more efficient performance, the negative brain-behavior correlation is expected to be associated with response inhibition. Figure 3A shows the within-subject correlation in the 3rd to 12th runs. Negative correlations were revealed in the anterior cingulate cortex (peak coordinate: −10, 4, 36; *t*_(44)_ = −4.5 at (10, 18, 30) from Neurosynth) and the cerebellum (lobule VIII; peak coordinate: −28, −52, −40; *t*_(44)_ = −4.4; Figure 3A, see Supplementary Figure S2 for whole-brain slices). Scatter plots in these two regions are shown in Figure 3B for one representative subject. To compare the negative correlation pattern of the across- and within-subject correlations, 10 common regions of interest were defined by averaging the normalized z-maps of the across- and within-subject correlations and detecting regions with 10 greatest z-scores. Although the z-scores of the correlation analyses depend on the data structure of the number of the subjects/runs, the present dataset exhibited greater negative correlation in the across-subject analysis than in the within-subject analysis (*t*_(9)_ = 3.1, *P* < 0.01; Supplementary Figure S3A). Alternatively, the common regions were defined based on independent dataset, using the coordinates reported in Chikazoe et al. (2009b), where the same authors used a similar version of the stop-signal task. Greater negative correlation in the across-subject analysis was similarly observed (*t*_(5)_ = 2.6, *P* < 0.05; Supplementary Figure S3B).

**Figure 4:**
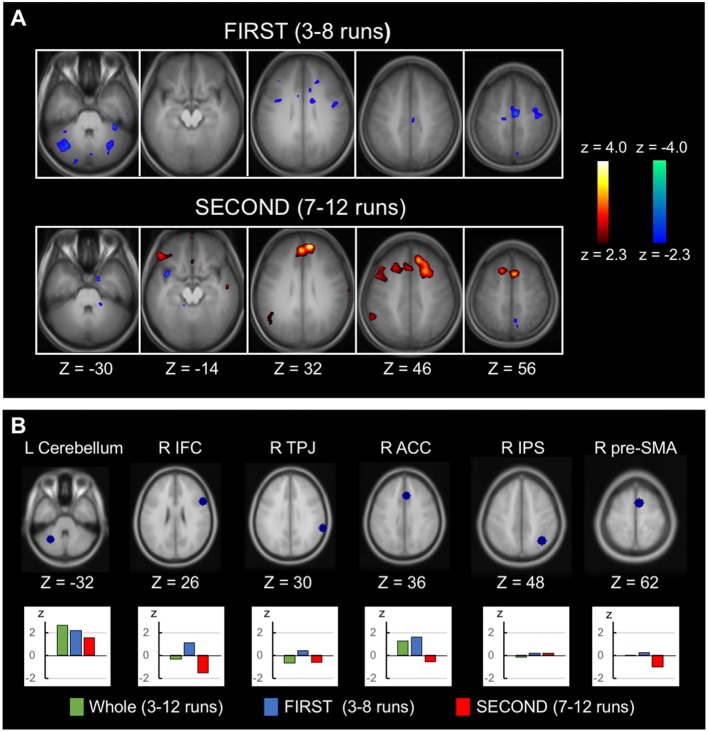
Time-related changes of the within-subject correlation. **(A)** Statistical maps of the within-subject (across-run) correlation between the SSRT and the brain activity related to response inhibition in FIRST (3rd to 8th) six runs and SECOND (7th to 12th) six runs. The format is similar to that in Figure 2C. **(B)** Regions of interest analyses of the temporal changes of the within-subject correlation, showing correlation in the whole runs, FIRST runs and SECOND runs. The coordinates were defined based on independent datasets from Chikazoe et al. (2009b).

Greater negative correlation associated with response inhibition in the latter half of the runs than in the earlier half was reported in Jimura et al. (2014) using the across-subject analysis. The temporal changes in the within-subject analysis was also examined in this study, analyzing FIRST (3rd to 8th runs) and SECOND (7th to 12th runs) parts of the runs. Figure 4A (top) shows the within-subject correlation for FIRST runs (see Supplementary Figure S4 for whole-brain slices). Negative correlations were dominant in the whole brain (*t*_(447.9)_ = −12.0, *P* < 0.001, the degrees of freedom corrected with the number of resels). Figure 4A (bottom) shows the within-subject correlation for SECOND runs (see Supplementary Figure S5 for whole-brain slices). Conversely, positive correlations were dominant in the whole brain (*t*_(487.5)_ = 5.0, *P* < 0.001). The difference between FIRST and SECOND did not reveal any significant correlation, based on the statistical procedures used in Figure 3A. For a comparison purpose, the across-subject correlations for FIRST and SECOND runs in whole-brain slices are shown in Supplementary Figures S6, S7. Regions of interest analyses were performed further, using the coordinates from independent dataset of Chikazoe et al. (2009b). Greater within-subject correlation in FIRST than SECOND was observed in the anterior cingulate region (*t*_(45)_ = 2.4, *P* < 0.05), whereas no correlation difference was observed in the cerebellar region (Figure 4B). Additionally, the within-subject correlation analysis was performed for Go RT, instead of SSRT. There was little within-subject correlation in the anterior cingulate or cerebellar regions (Supplementary Figure S8).

## Discussion

The present study employed the within-subject correlation analysis, calculating across-run correlation for each subject between the behavioral index and the brain activity associated with response inhibition. Within-subject correlation was observed in the anterior cingulate cortex and the cerebellum. Moreover, differential patterns of correlation were observed in the earlier vs. later runs. These results suggest that the within-subject correlation analysis complements the across-subject correlation analysis by revealing different aspects of cognitive/affective processes related to response inhibition.

This study examined both the across- and within-subject correlation analyses using the same data of 46 subjects, with 10 effective runs in each subject. There was a whole-brain level tendency that the across-subject negative correlation was greater than the within-subject correlation (Supplementary Figure S3), suggesting that the across-subject variability is greater than the within-subject variability. At the same time, the relative robustness of the correlation analyses depends on the data structure of the number of the subjects/runs, and it is possible that the within-subject negative correlation is more robust when more than 10 effective runs are collected for each subject. Because the latter runs exhibited whole-brain tendency of positive correlation (Figure 4A), however, collecting more than 10 runs may result in less robust negative correlation. Therefore, it is also possible that the number of runs in the present dataset is reasonable for the within-subject correlation analysis.

The across-subject correlation analysis reveals functional areas where more efficient performers with shorter SSRT elicit higher brain activity, whereas the within-subject (across-run) correlation analysis reveals functional areas where more efficient performance in a run in the same subject elicits higher brain activity. The within-subject correlation observed in the anterior cingulate cortex (Figure 3A) may reflect across-run fluctuation of monitoring processes (Carter et al., 1998; Botvinick et al., 2001; Braver et al., 2001) during performance of the stop-signal task that contributed to response inhibition. The correlation observed in the cerebellum may reflect motor/cognitive control processes (Imamizu et al., 2000; Ito, 2008) that has been observed in previous studies of the across-subject correlation analysis (Ghahremani et al., 2012; Jimura et al., 2014). Regions of interest analyses revealed that the anterior cingulate correlation was dominant in the earlier runs, whereas the cerebellar correlation was relatively constant (Figure 4B). The differential results suggest multiple mechanisms associated with inhibitory processes that fluctuate on a run-by-run basis, with the anterior cingulate mechanism contributing only in the earlier runs. The anterior cingulate activity is known to decline more rapidly than learning of attentional control in Stroop task, suggesting that the anterior cingulate cortex is involved in other aspects than implementation of top-down attentional control (Milham et al., 2003), such as monitoring processes (Carter et al., 1998; Botvinick et al., 2001; Braver et al., 2001). It has also been reported that the activity in the anterior insula/inferior frontal operculum network, to which the anterior cingulate cortex belongs, declines more slowly during sequential learning of new tasks, than other lateral frontal cortex networks (Hampshire et al., 2016). The results may raise the possibility that sequential learning of new tasks requires monitoring processes long after the tasks are learned, in order to inhibit proactive interference from previously acquired tasks.

Interestingly, the positive correlation was observed in the latter runs, primarily in the medial prefrontal cortex (Figure 4A), which is known as a part of a cognitive control network (Hu et al., 2016), or as a member area of the default-mode network (Fox and Raichle, 2007). It is unlikely that the brain activity related to cognitive control makes performance worse. Alternatively, subjects might have recruited more brain regions when they performed the task using a less-efficient strategy. Based on the function of the default mode network (Buckner et al., 2008), it is suggested that subjects were not focused on the external environment, which led to worse performance in latter runs.

Brain-behavior correlation changed during 10 runs of performance in the present study. While the across-subject analysis revealed enhanced negative correlation during the second vs. the first half of the runs (Jimura et al., 2014), the within-subject analysis revealed opposing correlations in FIRST and SECOND runs, showing negative and positive correlations in FIRST and SECOND runs, respectively (Figure 4A). Although the across-subject correlation has been used to identify robust functional areas, the within-subject correlation analysis may complement the across-subject analysis by shedding light on the cognitive/affective processes that fluctuate in a shorter period and may also contribute to rapid improvement of performance in athletes in the field of sports science (Nakata et al., 2010; Miyashita, 2016).

## Author Contributions

TY, AO, TO and SK designed the research and wrote the manuscript. TY, AO, TO, KJ and SK analyzed the data.

## Conflict of Interest Statement

The authors declare that the research was conducted in the absence of any commercial or financial relationships that could be construed as a potential conflict of interest.

## Footnotes

^1^www.fil.ion.ucl.ac.uk/spm

^2^https://fsl.fmrib.ox.ac.uk/fsl/fslwiki/Randomise

^3^http://neurosynth.org/analyses/terms/response%20inhibition/
