# Supplementary material for: Within-Subject Correlation Analysis to Detect Functional Areas Associated With Response Inhibition
Source: Front Hum Neurosci. 2018 May 22;12:208. doi: 10.3389/fnhum.2018.00208 (PMC5972214; doi:10.3389/fnhum.2018.00208)
Supplement: Supplementary file 1 [file Image_1.PDF]

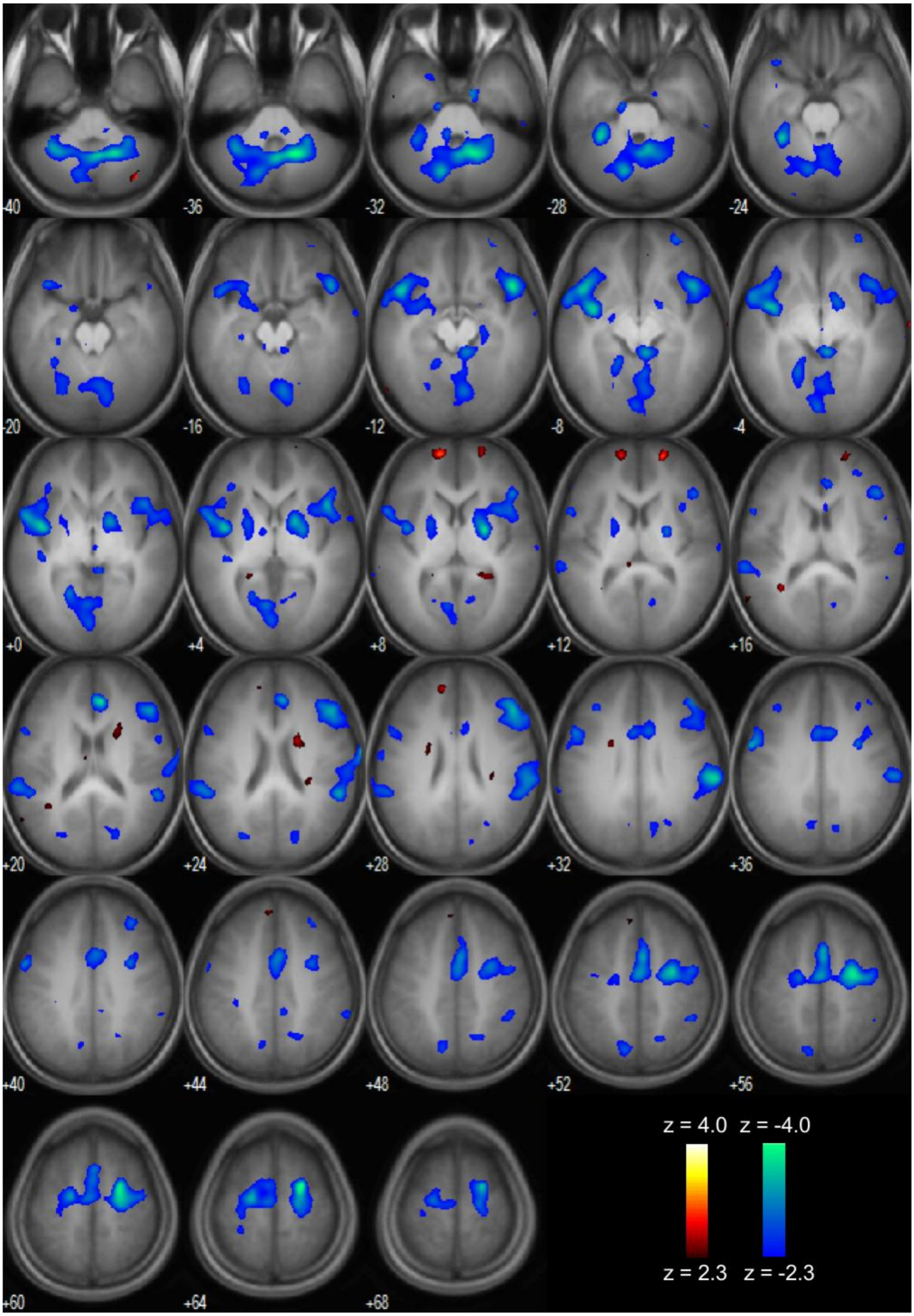

Figure S1 Whole-brain slices of statistical maps of the across-subject correlation (3rd to 12th).

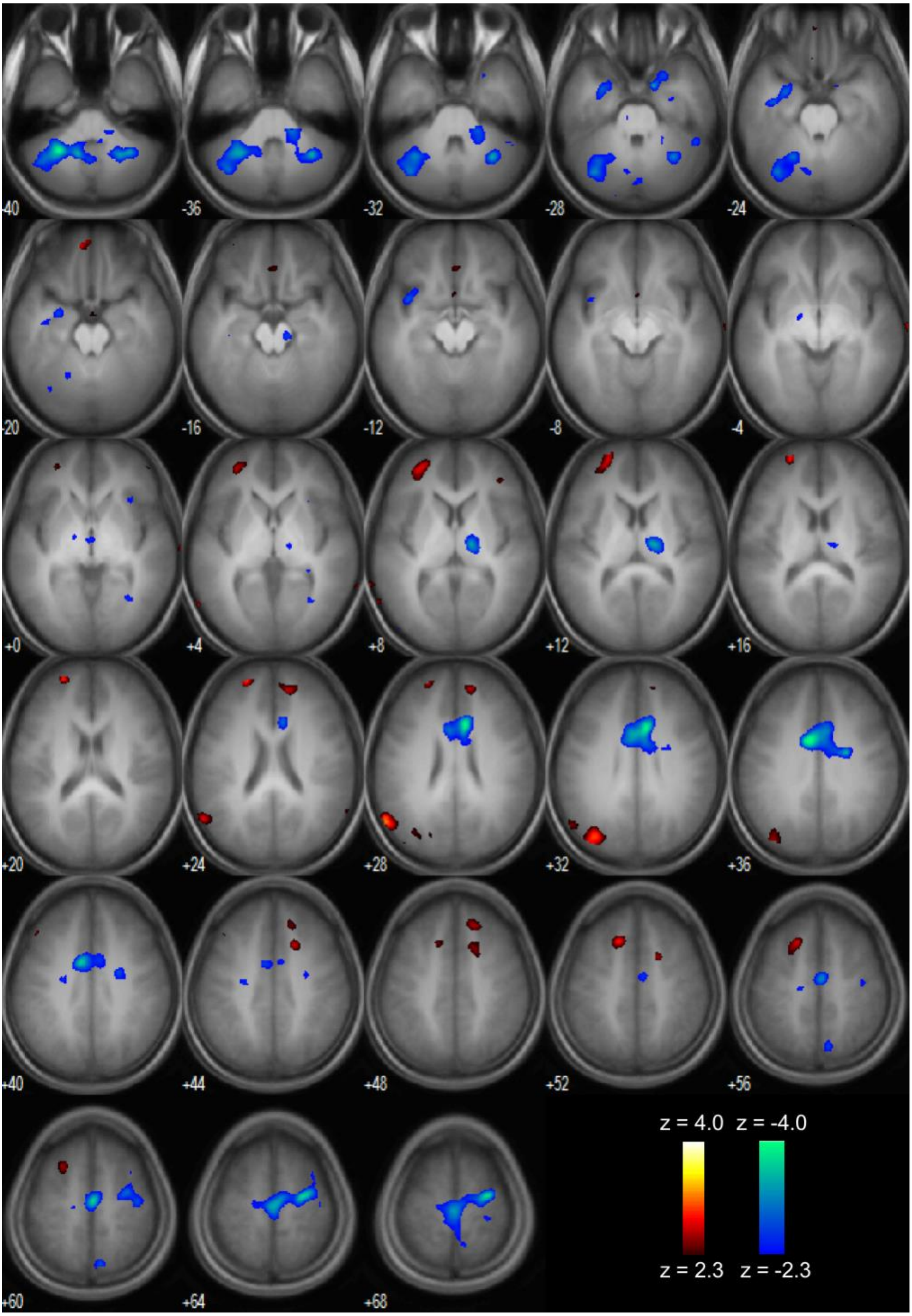

Figure S2 Whole-brain slices of statistical maps of the within-subject (across-run) correlation (3rd to 12th).

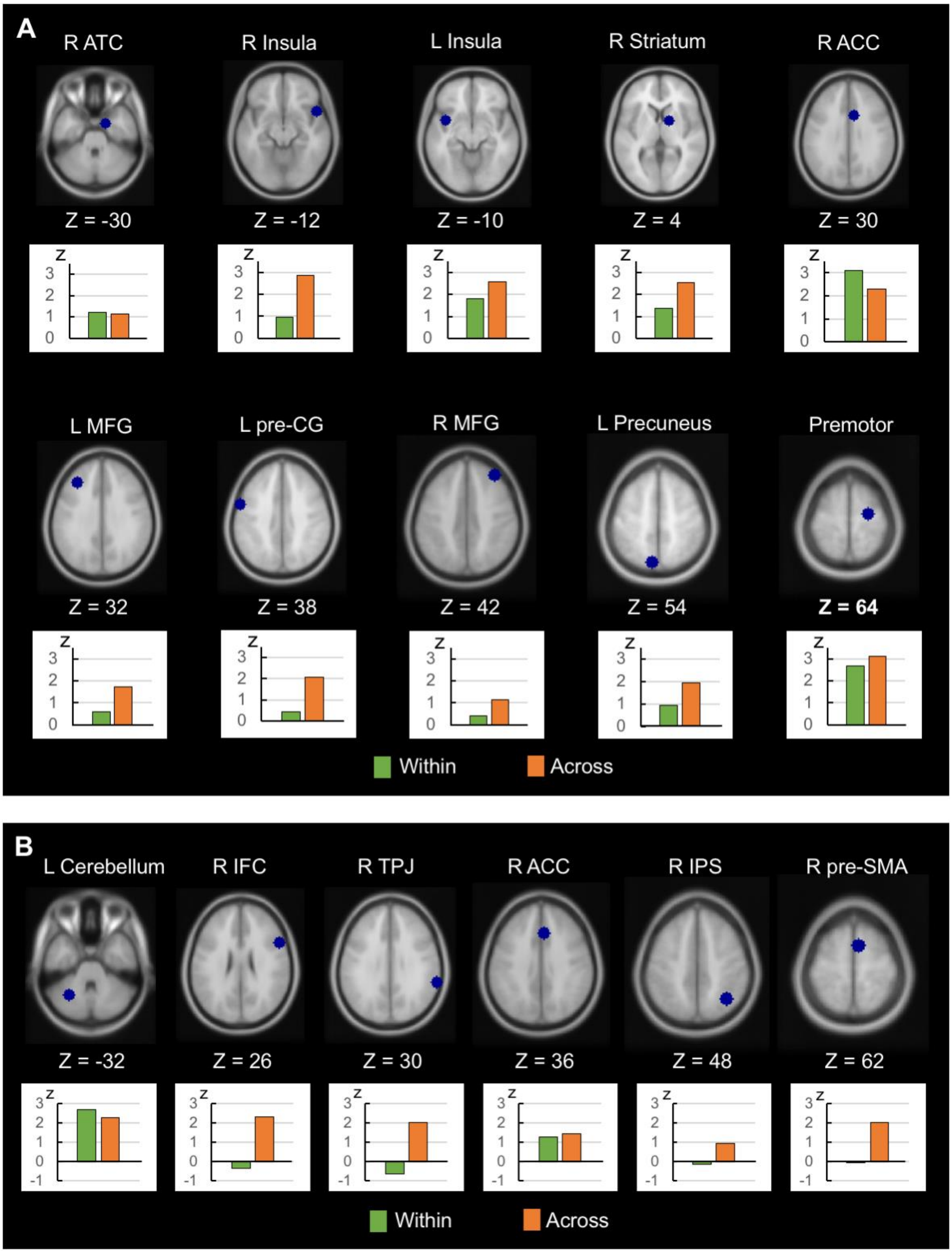

Figure S3 Comparison of statistical significance between the within- and the across-subject correlation. Negative z scores are reversed into positive values. (A) Ten regions of interest were defined by ten greatest z-scores from averaged normalized z-maps of the within- and across-subject correlations. (B) Six regions of interest were defined by the coordinate of Chikazoe et al. (2009b).

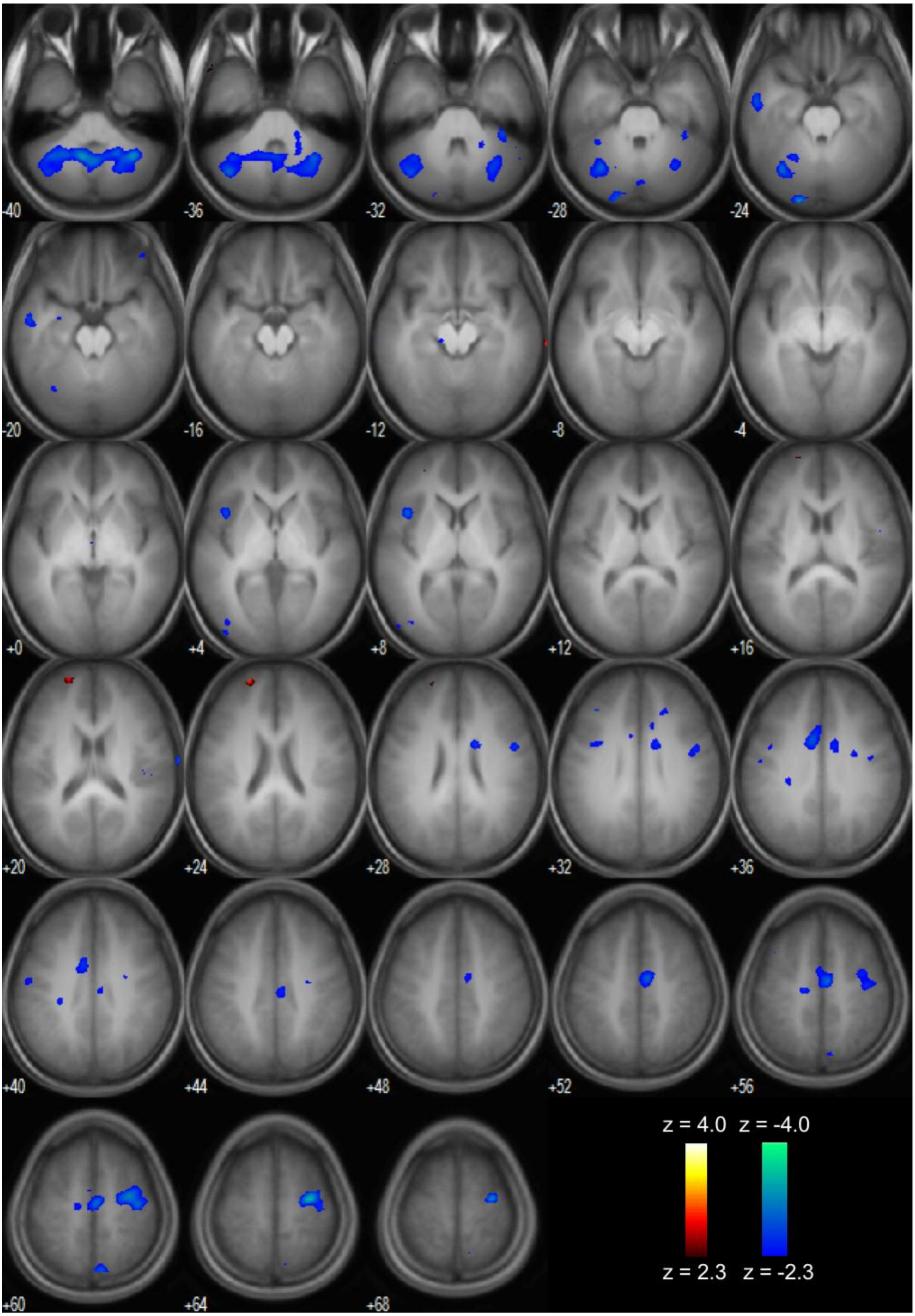

Figure S4 Whole-brain slices of statistical maps of the within-subject (across-run) correlation in FIRST runs (3rd to 8th).

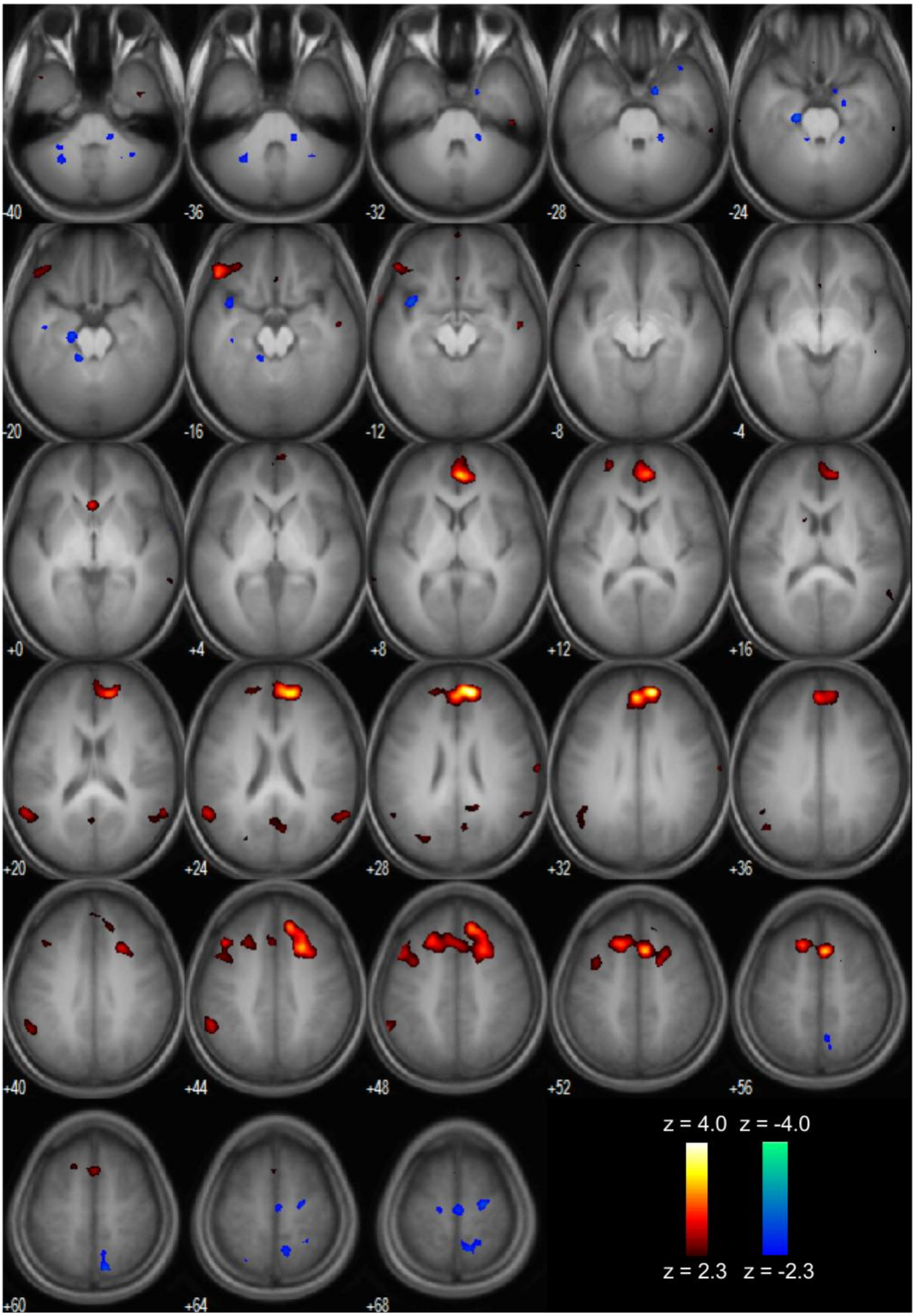

Figure S5 Whole-brain slices of statistical maps of the within-subject (across-run) correlation in SECOND runs (7th to 12th).

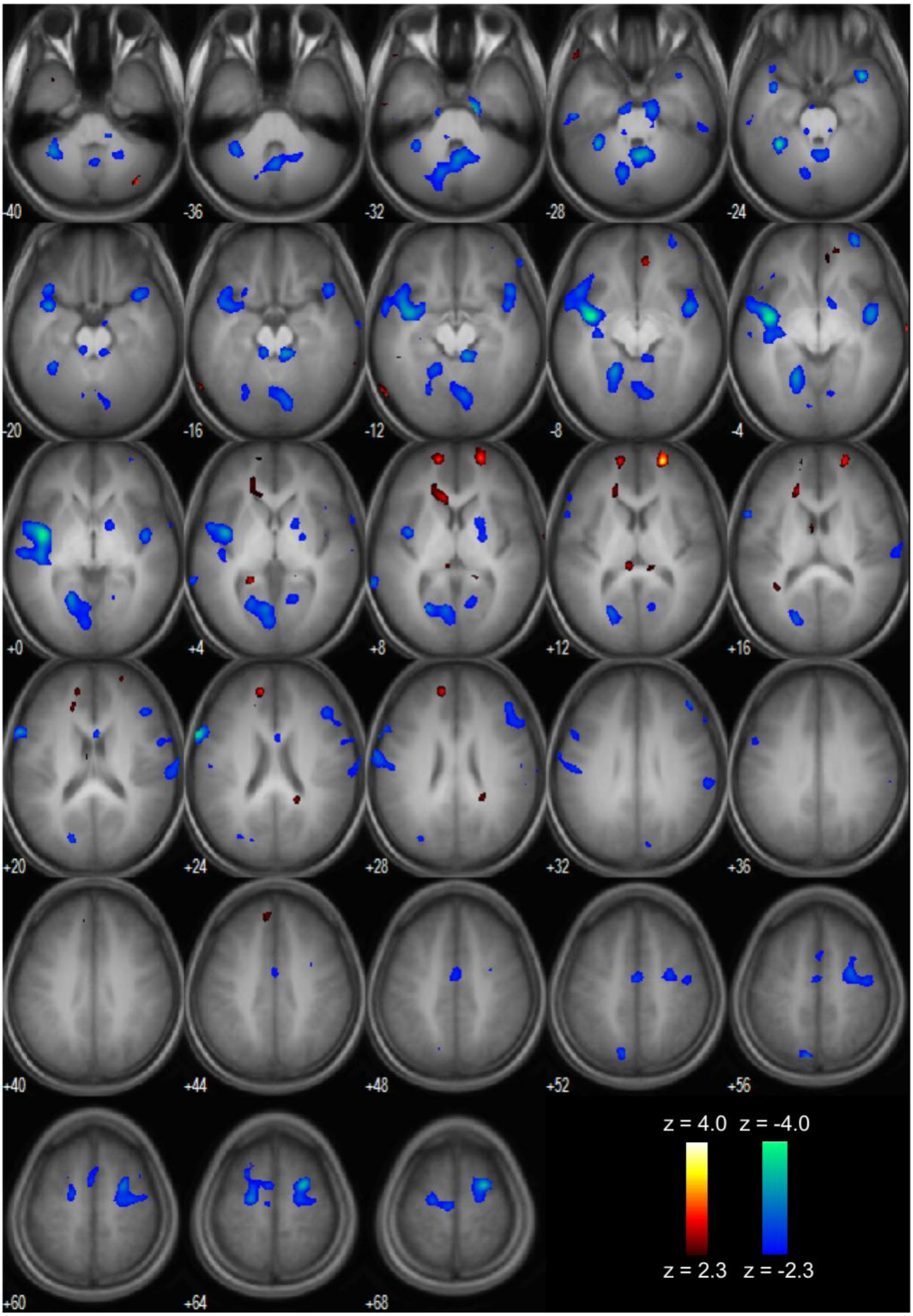

Figure S6 Whole-brain slices of statistical maps of the across-subject correlation in FIRST runs (3rd to 8th).

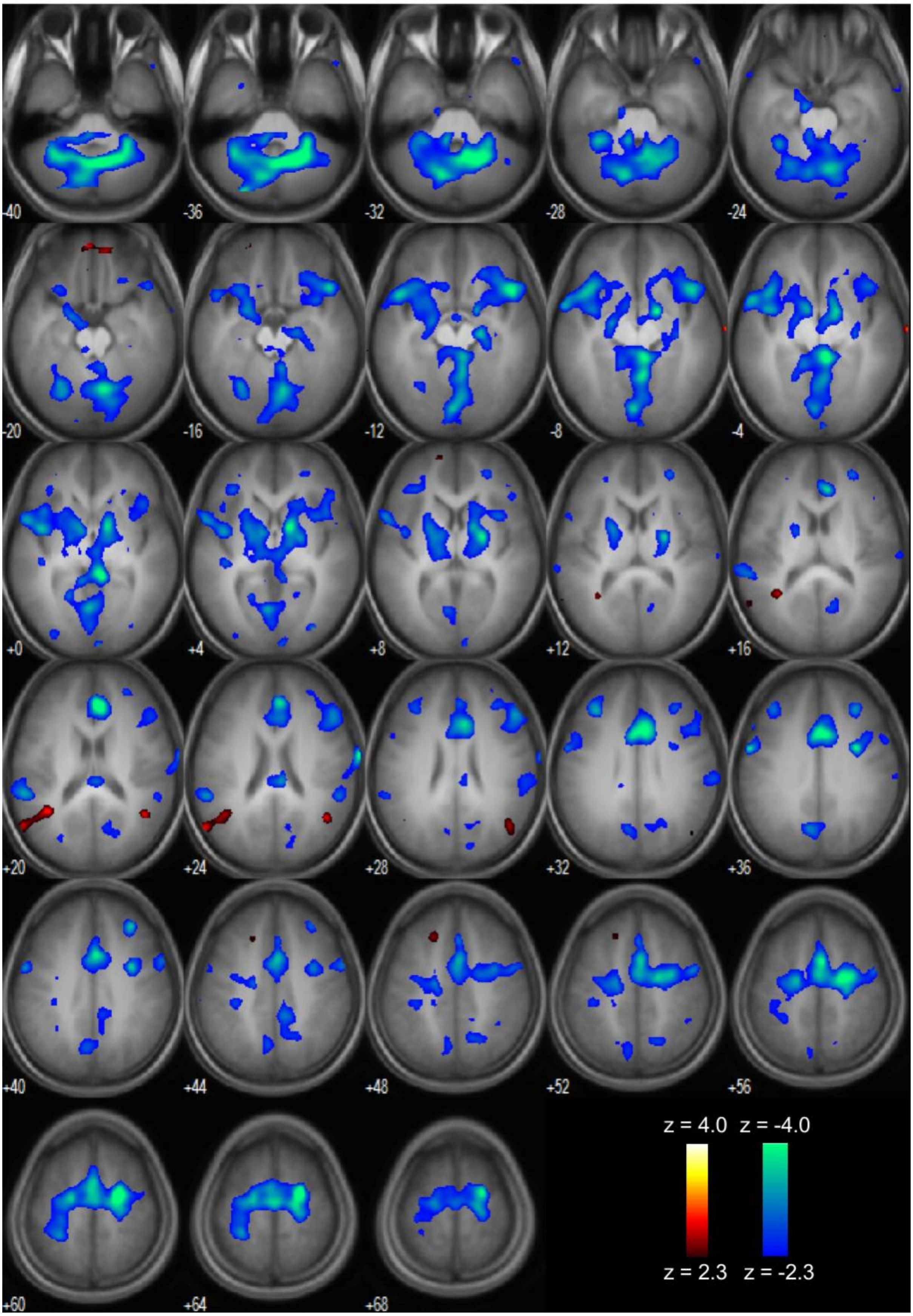

Figure S7 Whole-brain slices of statistical maps of the across- subject correlation in SECOND runs (7th to 12th).

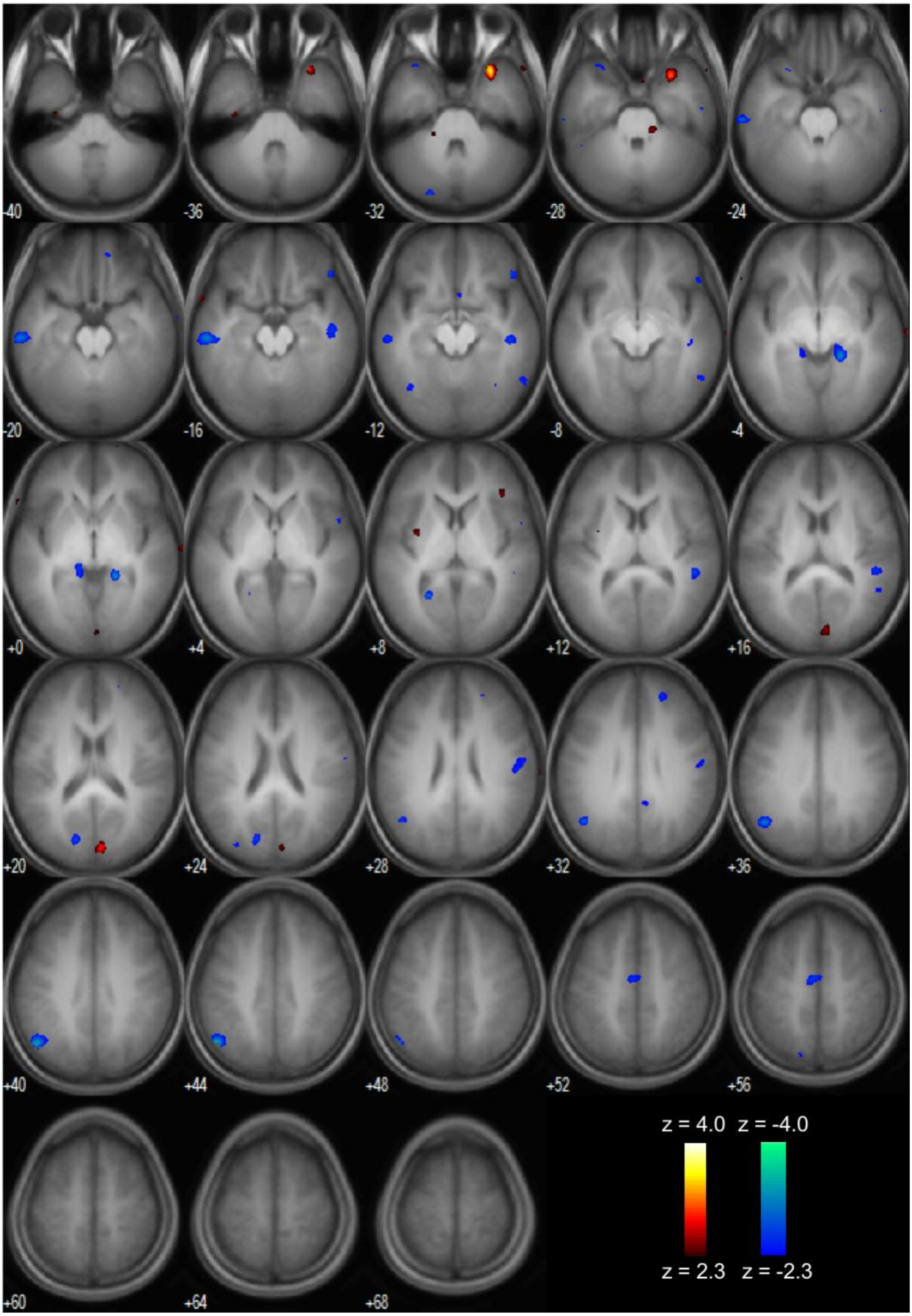

Figure S8 Whole-brain slices of statistical maps of the within-subject (across-run) correlation with Go-RT (3rd to 12th).
